# A Brief Report of Sexual Violence among Universities with NCAA Division I Athletic Programs

**DOI:** 10.3390/bs9020017

**Published:** 2019-02-04

**Authors:** Jacquelyn D. Wiersma-Mosley, Kristen N. Jozkowski

**Affiliations:** 1 School of Human Environmental Sciences, University of Arkansas, Fayetteville, AR 72701, USA; 2 The Department of Health, Human Performance and Recreation, University of Arkansas, Fayetteville, AR 72701, USA; kjozkows@uark.edu; 3 The Kinsey Institute for Research in Sex, Gender and Reproduction, Bloomington, IN 47405, USA

**Keywords:** NCAA Division I, violence against women, college campuses, Clery data

## Abstract

Violence against women on college campuses continues to be a pervasive public health problem with approximately one in five women experiencing sexual assault and one in nine women experiencing rape while in college. The current study examined relationship and sexual violence among National Collegiate Athletic Association (NCAA) Division I universities. Based on previous research, Division I universities seem to report higher rates of sexual assault, but within-group differences have yet to be examined. The data include 1422 four-year private and public institutions with at least 1000 students who submitted Clery data (2014) on rape, domestic and dating violence, and stalking. Division I campuses reported significantly higher reports of violence against women compared to Division II, III, and universities with no athletic programs. There were no differences in violence reported across the three subdivisions within Division I, however, certain conferences reported significantly higher relationship and sexual violence within the football bowl and football championship subdivisions. These findings have important implications for targeting higher risk campuses, such as the Big 10, Big 12, Ivy League, Pac-12, and SEC with much needed sexual assault prevention programs.

## 1. Introduction

Sexual violence on college campuses continues to be a pervasive public health issue with approximately one in five women experiencing sexual assault and one in nine women experiencing rape while in college [1]. Additionally, one in five students have experienced domestic violence with a current partner [2]; and one in five female students reported some form of stalking victimization [3]. Sexual violence is associated with negative mental health outcomes, including posttraumatic stress disorder (PTSD) [4,5], depression [6], anxiety [7,8], substance use disorders [7,8], and suicidal behavior [9]. Perhaps the most disconcerting part is that the prevalence of sexual violence on campuses has not changed in nearly sixty years [10,11]. In other words, this problem is by no means a new one. 

When examining factors related to sexual violence on college campuses, previous research has primarily assessed individual-level risk factors such as prior victimization (both sexual and physical violence) [12], substance use [13], age [14], ethnicity (i.e., White/Caucasian) [15], membership in a Greek organization [16,17], number of previous sexual partners [18], and attitudes and beliefs such as masculine ideology [2,18,19]. However, recent research suggests [20] that there are university level characteristics which may increase certain campuses’ propensity toward violence against women.

Research suggests that there are meaningful campus-level patterns that are associated with increased reporting of relationship and sexual violence. Using campus-level data (acquired via the Clery Act), four studies found that reports of sexual violence were higher on campuses with National Collegiate Athletic Association (NCAA) Division I athletic programs as compared to Division II, III and campuses with no athletics [21,22]. Additionally, using FBI data from 96 Division I campuses [23], researchers found that home football games increased reports of college-aged rape victimization by 58 percent compared to away football games which increased victimization by 15 percent. However, no studies, to which we are aware, have examined campus-level sexual violence specifically among Division I universities. 

Within the US, the NCAA [24] is the primary intercollegiate athletics governing body for varsity sports at four-year universities. There are approximately 1098 campuses that are affiliated with the three NCAA divisions: Division I schools generally have the largest student bodies (6000 teams, 170,000 student-athletes, 347 campuses); Division II is made up of 309 campuses, while Division III includes more than 180,000 student-athletes at 442 campuses. Division I is subdivided into the Football Bowl Subdivision (FBS), Football Championship Subdivision (FCS), and Non-football, Multi-sport. The subdivisions apply only to football (FBS, FCS) and the third group is considered simply Division I. FBS schools are generally more competitive, have larger budgets and student enrollment, and compete for the football BCS title (e.g., Bowl Championship Series). FBS programs tend to attract higher caliber players, have more players drafted to the National Football League (NFL), give more scholarships to players, and are more likely to have games televised [23].

With the current culture involving sexual assault allegations among high profiled universities (i.e., Baylor University, Michigan State University), it is imperative to examine the rates of sexual assault and relationship violence because Division I programs are considered to be at highest risk for violence. Previous research suggesting a link between Division I campuses and sexual assault, however, does not specifically assess violence within Division I programs. This study aims to address this gap in the literature by (1) examining the extent to which reported campus violence (inclusive of rape, domestic and dating violence, and stalking) differs across NCAA Divisions, and (2) examining potential differences in reported violence within Division I programs. 

## 2. Methods

Data for the current study were derived from the Campus Safety and Security Data Analysis Cutting Tool [25] provided by the Office of Postsecondary Education of the U.S. Department of Education, required by the Jeanne Clery Disclosure of Campus Security Policy and Campus Crime Statistics Act (Clery Act), the Higher Education Opportunity Act (2008), and the Violence Against Women Act (VAWA) [26]. The data included 1422 public or private-nonprofit four-year institutions with a student body of at least 1000. It is important to note that the crime data reported by the institutions have not been subjected to independent verification by the U.S. Department of Education. Therefore, the Department cannot vouch for the accuracy of the Clery data. In addition, Clery statistics may not capture actual reports of campus crime, as too often crimes go unreported, especially when it comes to crimes of violence against women [27]. Institutional Review Board approval was deemed unnecessary as all data were archival. Each university’s participation in intercollegiate sports was obtained from the NCAA [8]: no affiliation (i.e., no athletic programs; *n* = 384, 27%), Division III (397, 28%), Division II (296, 20%) and Division I (345, 24%). 

### Measures

All analyses controlled for *student enrollment* (ranging from 1000 to 66,046; *M* = 7970.93, *SD* = 9764.17), and campus reported *liquor violations* (ranging from 0–2639 violations; *M* = 126.23, *SD* = 218.14), via Clery. Liquor violations was used to assess the “party culture” of college campuses [20] and has been found to be significantly higher in Division I football programs [23]. 

Campus violence was assessed by examining the number of reported Clery crimes for rape, domestic and dating violence, and stalking. *Rape* is defined as the penetration, no matter how slight, of the vagina or anus, with any body part or object, or oral penetration by a sex organ of another person, without the consent of the victim. *Domestic violence* is defined as a felony or misdemeanor crime of violence committed, by a current or former spouse or intimate partner of the victim, by a person with whom the victim shares a child in common, by a person who is cohabitating with, or has cohabitated with, the victim as a spouse or intimate partner, by a person similarly situated to a spouse of the victim under the domestic or family violence laws of the jurisdiction in which the crime of violence occurred, or by any other person against an adult or youth victim who is protected from that person’s acts under the domestic or family violence laws of the jurisdiction in which the crime of violence occurred. *Dating violence* is defined as violence committed by a person who is or has been in a social relationship of a romantic or intimate nature with the victim…based on the reporting party’s statement and with consideration of the length of the relationship, the type of relationship, and the frequency of interaction between the persons involved in the relationship; this includes, but is not limited to, sexual or physical abuse or the threat of such abuse and does not include acts covered under the definition of domestic violence. *Stalking* is defined as engaging in a course of conduct directed at a specific person that would cause a reasonable person to fear for the person’s safety or the safety of others; or suffer substantial emotional distress. 

## 3. Results

First, we compared violence variables across NCAA Divisions I, II, and III, as well as campuses without athletic programs (four groups) via multivariate analysis of covariance (controlling for student enrollment and liquor violations). Findings indicated an overall significant difference across the four groups (Wilks’ Lambda = 0.89, *p* < 0.001). Based on univariate *F* tests and mean differences, Division I reported significantly higher violence compared to Division II, Division III and campuses with no athletics, as shown in Table 1. 

To further assess violence, we subdivided the Division I conferences based on their football sponsorship designation (refer to Table 2): Football Bowl Subdivision (*n* = 123, 36% campuses), Football Championship Subdivision (*n* = 126, 37% campuses) and Non-football, Multi-sport (*n* = 96, 28% campuses). We compared violence variables across these three groups using multivariate analysis of covariance, controlling for student enrollment and liquor violations, and found no significant group effect across the three subdivisions (Wilks’ Lambda = 0.99, *p* = 0.83). 

Given this somewhat surprising result, we then compared within-group differences by comparing all the conferences within each of the football sponsorship subdivisions (again, controlling for student enrollment and liquor violations). First, within the Football Bowl Subdivision, there was an overall significant group effect (Wilk’s Lambda = 0.59, *p* < 0.01), with significant group differences in dating violence only (*F* = 3.17, *p* = 0. 002; pairwise comparisons are noted in Table 1). The American Athletic conference (AA) reported a significantly higher number of dating violence crimes compared to the ACC, Mountain West, Pac-12, SEC, and Sun Belt conference. Second, within the Football Championship Subdivision, there was an overall significant group effect (Wilk’s Lambda = 0.32, *p* < 0.001), with significant group differences in rape (*F* = 10.52, *p* = 0.001), domestic violence (*F* = 2.02, *p* = 0.033), and stalking (*F* = 2.96, *p* = 0.002; all pairwise comparisons are noted in Table 1). For example, the Ivy League universities reported a significantly higher number of rape (21.80 on average) and stalking (8.78 on average) crimes as compared to all the other conferences within this subdivision. In addition, the Big Sky conference reported a significantly higher number of domestic violence crimes compared to all the other conferences. Last, there were no within-group differences for Non-football, Multi-sport Division I.

## 4. Discussion

The current study contributes to the growing body of literature by examining sexual and relationship violence across NCAA Divisions and, more specifically, within Division I schools as these campuses seem to be at greater risk for sexual violence [21,22,28]. Consistent with previous work, we also found that when compared to Division II and III campuses and campuses without an NCAA affiliation, Division I campuses reported higher sexual and relationship violence. Interestingly, there were no group differences across the three subdivisions within Division I. This was somewhat surprising given that the third group consisted of universities without football programs and research suggests that the majority of athletes accused of perpetrating sexual assault come from prominent football teams [28,29] and football game outcomes seem to be associated with increased sexual violence [23]. 

With regard to college sexual and relationship violence, underreporting is common [27,30]. As such, it may be the case that the incidences of the violence variables assessed in this study are indeed occurring at higher rates on campuses with football Division I programs, but they are not being reported and therefore not accounted for in the Clery data. Alternatively, perhaps there are other sports teams (e.g., basketball; lacrosse) at non-football program universities that generate the same response from the student body which increased risk for sexual assault perpetration that football games generate at Football Bowl and Football Championship campuses [23]. This could explain why we did not see group differences. It may be the case that we found no differences because we assessed a range of relationship violence variables as previous research has only assessed sexual violence. Finally, it is important to note that Clery data likely provides a very *conservative* estimate of campus violence. 

Victims’ reasons for not reporting are dominated by beliefs that others, especially those in positions of authority, will not believe them [31]. When less than 10% of victims report their assault [32], it is important that campuses encourage faculty, staff, and administrators to welcome reporting and to take reports seriously. In addition, campuses should also continue to increase their campus climate surveys, and make these data available to the public, so that it might increase awareness for students and their parents. A recent study [33] found that only half of Title IX coordinators had completed a campus climate survey, thus there is a lack of knowledge on the part of schools when they fail to survey their students. Increasing awareness may provide new practical insights that could shape decisions made by campus officials, healthcare workers, and reporting structures. 

In addition, research should examine whether Division I status affects the reporting of campus crimes. Universities with such high status athletic programs may be perceived to have more at stake in the face of public and media scrutiny. Pressure from athletics departments, administration, or even the surrounding community, whether intentional or unintentional, for victims not to report when the perpetrator is a high-status athlete could also explain why we did not see group differences across the subdivisions among Division I schools. Athletes who are part of well-known sports programs may receive perks such as power and prestige, which, in turn, can result in universities “looking the other way” or making exceptions when such individuals are accused of sexual or relationship violence. A recent study [34] presented a theoretical model based on empirical findings demonstrating how university systems perpetuate rape culture by allowing privileged and powerful groups (e.g., fraternities) disproportional power on campus. It may be the case that a similar model exists with athletes as the privileged group. For example, the Ivy League conference reported over 20 campus rapes, on average, in 2014 and was significantly higher in relationship violence compared to other conferences. Perhaps, the higher reports of violence occurring at Ivy League campuses is related, in some way, to entitlement, power and/or prestige of student-athletes at these universities. However, continued research is needed to further elucidate the association among power, prestige, entitlement and athletics to determine whether there would be support for such a model among athletes, in particular high-profile sports. Overall, it is important to empirically assess the extent to which reporting may be uniquely impacted at certain universities. 

In conclusion, the current study examined within-group differences in sexual and relationship violence among Division I-affiliated universities. These findings have important implications for targeting higher risk campuses within Division I, such as the Big 10, Big 12, Ivy League, Pac-12, and SEC, with much needed violence prevention programs for students, staff, faculty and administrators. 

## Figures and Tables

**Table 1:** Relationship violence across National Collegiate Athletic Association (NCAA) divisions.

	*N*	Rape	Domestic Violence	Dating Violence	Stalking
Divsion I campuses	345	4.26	2.29	2.47	3.41
Division II campuses	296	2.19	1.18	2.87	1.81
Division III campuses	397	3.40	1.11	2.02	2.06
Non-NCAA campuses	384	1.32	0.73	0.93	1.31
*F*		30.51 *	12.29 *	15.36 *	15.69 *

Note: All analyses control for student enrollment and liquor violations. * *p* < 0.001.

**Table 2:** Relationship violence within NCAA division I subdivisions.

	*n*	Rape	Domestic Violence	Dating Violence	Stalking
*Football Bowl*		6.85	3.60	3.79	5.44
American Athletic	11	11.65	1.84	9.38 ^abcde^	8.32
Atlantic Coast (ACC)	15	11.49	2.62	4.98 ^af^	6.21
Big 10	14	10.11	8.75	6.15 ^g^	12.59
Big 12	10	8.12	6.15	5.84 ^h^	10.86
Conference USA	14	7.41	3.05	7.82 ^ijk^	6.93
Mid-American	12	8.74	4.41	7.75 ^lmn^	6.53
Mountain West	10	7.72	5.75	2.86 ^bil^	9.11
Pac-12	12	10.06	6.12	0.47 ^cfghjm^	8.23
Southeastern (SEC)	14	6.77	6.84	3.70 ^dkn^	7.10
Sun Belt	11	4.32	3.11	4.56 ^e^	3.16
*F*		1.15	0.90	3.17 **	1.24
*Football Championship*		5.82	2.77	3.69	4.88
Big Sky	12	4.02 ^a^	4.97 ^abcdefghi^	−0.17	0.57 ^abc^
Big South	11	3.35 ^b^	0.36 ^aj^	2.08	2.99 ^d^
Colonial Athletic	10	6.66 ^cdef^	0.20 ^k^	1.67	3.10 ^e^
Ivy League	8	21.80 ^abcghijklmn^	3.39 ^bjk^	3.95	8.78 ^adefghijk^
Mid-Eastern Athletic	13	1.67 ^dgo^	2.46 ^c^	4.20	1.96 ^fl^
Missouri Valley FB	10	4.50 ^h^	1.37 ^d^	3.72	6.41 ^blmnop^
Northeast	9	2.13 ^i^	0.57 ^e^	1.46	2.07 ^gm^
Ohio Valley	12	3.05 ^j^	2.30 ^f^	2.50	2.13 ^hn^
Patriot League	8	6.7 5^kopq^	0.88 ^g^	3.27	5.73^c^
Southern	10	3.58 ^l^	1.63 ^h^	3.45	2.78 ^io^
Southland	13	1.25 ^emp^	1.72 ^i^	2.45	2.31 ^jp^
Southwestern Athletic	10	1.25 ^fnq^	2.62	3.99	2.95 ^k^
*F*		10.52 ***	2.02 *	1.50	2.96 **
*Non-football, multi-sport*		*5.13*	*3.34*	*3.40*	*4.97*
American East	9	8.07	4.44	5.67	5.52
Atlantic Sun	8	6.86	0.81	2.09	0.99
Atlantic 10	14	4.99	3.49	3.44	6.72
Big East	10	3.41	2.27	1.48	3.27
Big West	9	2.78	5.49	3.87	5.35
Horizon	10	4.08	2.73	2.45	2.79
Metro Atlantic	11	4.59	0.69	2.03	1.94
The Summit League	9	3.39	2.91	3.91	5.48
West Coast	10	3.93	1.12	0.79	3.56
Western Athletic	6	1.89	4.90	1.76	2.72
*F*		1.51	1.18	1.74	0.98

Note: All analyses control for student enrollment and liquor violations. Pairwise comparisons (matching letters) indicate within-group differences within each of the subdivisions. * *p* < 0.05, ** *p* < 0.01, *** *p* < 0.001.
